# Clinical Outcomes of Ceftazidime–Avibactam versus Ceftolozane–Tazobactam in Managing Pseudomonal Infections in Patients Undergoing Renal Replacement Therapy

**DOI:** 10.3390/antibiotics13080699

**Published:** 2024-07-26

**Authors:** Wasim S. El Nekidy, Mooza Al Ali, Emna Abidi, Rania El Lababidi, Diaa Alrahmany, Islam M. Ghazi, Mohamad Mooty, Fadi Hijazi, Muriel Ghosn, Jihad Mallat

**Affiliations:** 1 Cleveland Clinic Abu Dhabi, Abu Dhabi P.O. Box 112412, United Arab Emirates; alalim6@clevelandclinicabudhabi.ae (M.A.A.); abidiee@clevelandclinicabudhabi.ae (E.A.); ellabar@clevelandclinicabudhabi.ae (R.E.L.); mootym@clevelandclinicabudhabi.ae (M.M.); hijazif@clevelandclinicabudhabi.ae (F.H.); ghosnm@clevelandclinicabudhabi.ae (M.G.); mallatj@clevelandclinicabudhabi.ae (J.M.); 2 Cleveland Clinic Lerner College of Medicine, Case Western Reserve University, Cleveland, OH 44195, USA; 3 Pharmaceutical Care Department, Directorate General of Medical Supplies, Ministry of Health, Muscat 393/100, Oman; diaaeldin@moh.gov.om; 4 Arnold and Marie Schwartz College of Pharmacy, Long Island University, Brooklyn, NY 11201, USA; islam.ghazi@liu.edu

**Keywords:** ceftazidime–avibactam, ceftolozane–tazobactam, dialysis, outcomes

## Abstract

The optimal doses of ceftazidime–avibactam (CZA) and ceftolozane–tazobactam (C/T) for treating multidrug-resistant (MDR) Pseudomonas aeruginosa (PSA) in patients utilizing renal replacement therapy (RRT) are not well established. Hence, the objective of this study is to evaluate the clinical outcomes associated with the suggested doses of CZA and C/T in patients with PSA infection utilizing RRT. Methods: This is a retrospective study conducted at our hospital between September 2018 and March 2022. Clinical cure was the primary endpoint, while microbiologic cure, 30-day recurrence, and 30-day mortality were the secondary endpoints. Results: In total, 45 subjects met the inclusion criteria, with 25 receiving CZA and 20 receiving C/T. The median age was 69 (52–81) and 69 (61.5–83) years, respectively, while the median weight was 70 (55.5–81.5) and 66 (57–79) kg, respectively. Clinical cure was achieved in 12 (48%) subjects in the CZA group and 12 (60%) in the C/T group (*p* = 0.432). Of the 36 subjects who had repeated cultures, a microbiologic cure was achieved in 14/23 (60%) subjects and 10/13 (76.9%) subjects (*p* = 0.273). Thirty-day recurrence was reported in 3 (12%) cases in the CZA group and 6 (30%) in the C/T group (*p* = 0.082). The 30-day mortality was 13 (52%) subjects in the CZA group and 10 (50%) in the C/T group (*p* = 0.894). The median maintenance dose of CZA was 1.88 (0.94–3.75) g and 2.25 (1.5–2.25) g for C/T. Multivariate logistic regression analysis indicated that both drugs did not differ significantly in clinical cure. Bloodstream infection (BSI) (OR = 25, 95% CI: 1.63–411.7, *p* = 0.021) was the only independent factor associated with clinical cure in this population. Conclusions: Our findings indicated that C/T and CZA did not significantly differ in achieving clinical cure in patients with MDR PSA infections undergoing RRT. Larger clinical trials are needed to confirm our findings.

## 1. Introduction

Infections caused by multidrug-resistant (MDR) *Pseudomonas aeruginosa* (PSA) present a significant therapeutic challenge due to the limited availability of effective antibacterial agents. Ceftazidime–avibactam (CZA) and ceftolozane–tazobactam (C/T) are two novel combinations of cephalosporin and beta-lactamase inhibitors that have shown clinical efficacy against MDR PSA in clinical trials [1,2,3,4,5,6].

Both CZA and C/T are primarily eliminated by the kidneys. Consequently, in patients with compromised kidney function, these drugs can accumulate due to their prolonged half-life, necessitating lower doses than the standard [7]. Additionally, both drugs have low protein binding capacities, making them readily available for clearance by renal replacement therapy (RRT) [4,8,9,10,11].

At our institution, our dosing strategy involves administering a full dose (loading dose) of CZA (2.5 g every 8 h) or C/T (1.5 to 3 g every 8 h) intravenously (IV) for the first 24 h, followed by a maintenance dose adjusted according to the RRT modality [12]. For example, for patients undergoing continuous veno-venous hemofiltration (CVVH), the recommended maintenance doses for CZA range from 0.94 to 1.25 g IV every 8 h and for those undergoing intermittent hemodialysis (IHD), the recommended maintenance doses for CZA range from 0.94 g to 1.25 g IV every 24 h. Similarly, for patients on CVVH, the recommended maintenance dose for C/T is 1.25 g IV every 8 h and for those on IHD, the recommended maintenance dose for C/T is 0.75 g IV every 8 h [10,11].

It is advisable to maintain a 4:1 ratio of CZA when reducing the doses in patients with compromised kidney function due to the linear pharmacokinetics of this combination [7]. Similarly, C/T exhibits linear pharmacokinetics in patients with normal renal function but prolonged half-lives in patients with compromised kidney function [4,8].

The optimal doses of CZA and C/T in patients with PSA infections utilizing RRT are still not well established. Furthermore, clinical outcome data using the suggested doses of both drugs in this population are limited to case reports. Hence, the goal of this study was to compare the clinical outcomes of the dosing regimens of CZA versus C/T at our institution in patients with PSA infection utilizing RRT.

## 2. Results

In total, 45 patients met the inclusion criteria, with 25 receiving CZA and 20 receiving C/T. Baseline characteristics are presented in Table 1. Among the CZA recipients, nine (36%) were males, compared to seven (35%) in the C/T group, with median ages of 69 (52–81) years and 69 (61.5–83) years, respectively. The median weight was 70 (55.5–81.5) kg in the CZA group and 66 (57–79) kg in the C/T group. Of the 25 subjects who received CZA, 19 (76%) were diagnosed with pneumonia, compared to 17 (85%) in the C/T group. No significant differences in the baseline characteristics were observed between the two groups (Table 1).

Clinical cure was achieved in 12 (48%) subjects in the CZA group and 12 (60%) in the C/T group (*p* = 0.432). Most PSA organisms were carbapenem-resistant, with 18 (75%) in the CZA group and 18 (90%) in the C/T group (*p* = 0.187). Additionally, 15 (60%) subjects in the CZA group received concomitant antibiotics, compared to 14 (70%) in the C/T group (*p* = 0.486). Clinical outcomes are detailed in Table 2.

In total, 18 (72%) subjects in the CZA group and 15 (75%) in the C/T group received a loading dose. The total median daily loading dose was 3.75 (2.5–1.5) g for CZA and 4.5 (2.25–4.5) g for C/T. The median maintenance dose was 1.88 (0.94–3.75) g for CZA and 2.25 (1.5–2.25) g for C/T. The most common doses were 1.25 g IV daily for CZA in IHD patients and 1.25 g IV every 8 h for CVVH patients. For C/T, the doses were 0.75 g IV every 8 h for IHD and 1.5 g IV every 8 h for CVVH.

Among the 36 subjects with repeated cultures, a microbiologic cure was achieved in 60% with CZA and 76.9% with C/T (*p* = 0.273). The median APACHE-IV score was not significantly different between the CZA and C/T groups (94 vs. 87, *p* = 0.562) among the 32 subjects admitted to the ICU. The median MIC of PSA to CZA was 1.5 mcg/mL (0.875–7) in the 24 subjects receiving the drug and 1 mcg/mL (0.75–2) in the 12 patients receiving C/T.

In the CZA group, of the 15 patients (60%) in the C/T group, 14 patients (70%) received concomitant antibiotics, primarily tobramycin, colistin, and gentamicin. Inhaled antimicrobials were used by 6 subjects receiving concomitant antibiotics. Specifically, among those receiving concomitant antibiotics, six patients in the CZA group received colistin compared to none in the C/T group, one received gentamicin in the CZA group versus three in the C/T group, and two received tobramycin in the CZA group compared to seven in the C/T group. There were no documented central nervous system toxicities with the utilized doses. The multivariate logistic regression analysis showed that bloodstream infection (BSI) (OR = 25, 95% CI: 1.63–411.7, *p* = 0.021) was the only independent factor associated with clinical cure among the clinically relevant variables (Table 3).

## 3. Discussion

Our findings indicate that patients with BSI treated using either CZA or C/T had greater odds of achieving a clinical cure when compared to patients with pneumonia. On the other hand, the model did not indicate that the drug used (CZA and C/T), concomitant antibiotics, loading dose, maintenance dose, or therapy duration were independent predictors of clinical cure (Table 3).

Our findings indicated that BSI is more likely to be treated when compared to pneumonia in this population. This finding suggests that the doses utilized in this study could have been sufficient in managing BSI secondary to MDR PSA, while higher doses might be needed for pneumonia with the same bacteria. Furthermore, the use of concomitant antimicrobials (aminoglycosides or colistin), regardless of their susceptibility to the reported PSA isolates, did not show synergistic mechanisms in these settings.

To date, no head-to-head clinical outcome studies have compared CZA to C/T in patients with MDR PSA infections undergoing RRT. Although, in a large retrospective multicenter study conducted in six tertiary centers in Saudi Arabia, Almangour et al. compared 30-day mortality, clinical cure, and safety outcomes of C/T versus CZA in managing MDR PSA in 200 patients, 56% of whom were in critical care units. Additionally, 41% of the patients received combination therapy. The multivariate regression analysis, adjusted for confounders, did not reveal any significant differences in mortality, clinical cure, or acute kidney injury [13]. Furthermore, in a literature review by Aviles Martinez et al. aimed at identifying whether C/T or CZA would be more effective in clinically curing adults with complicated intra-abdominal infections secondary to MDR PSA and in reducing mortality, the authors concluded that there was no significant difference between the two drugs [14]. Although our findings are similar to the aforementioned studies, we are the first to compare both drugs in patients undergoing RRT. Moreover, we are evaluating the clinical efficacy of the recommended dosing utilized at our institution in this population.

Several microbiologic studies have compared the in vitro susceptibilities of both agents against PSA. For example, Hirsch et al. measured the minimum inhibitory concentrations (MICs) of CZA vs. C/T against 60 PSA isolates using the broth microdilution method [13]. They found that both agents had over 80% activity against MDR PSA but that the MICs for C/T were four-fold lower compared to CZA, suggesting enhanced in vitro activity of C/T in these infections [15]. Similarly, Alatoom et al., using Etest strip MIC, found that the activity of both drugs against 30 MDR PSA isolates was comparable, with 94% susceptible to CZA (MIC50: 1.5 mg/mL) and 97% susceptible to C/T (MIC50: 0.75 mg/mL) [16]. They concluded that the MIC of C/T was lower than CZA MIC, indicating improved in vitro activity of the former. Consistent with these findings, our results showed that the median MIC of C/T against MDR PSA isolates was lower than that of CZA. However, there was no significant difference in both microbiologic and clinical cures when comparing the two drugs.

Pharmacokinetic studies for both Ceftazidime–avibactam (CZA) and Ceftolozane–tazobactam (C/T) in patients using renal replacement therapy (RRT) are limited [10,11]. The pharmacokinetics/pharmacodynamics of beta-lactams require that the free drug plasma level exceeds the minimum inhibitory concentration (MIC) of the infecting microorganism (*f*T>MIC). It is suggested that the percentage of *f*T>MIC should be 100% in critically ill patients [17,18].

The FDA-approved dosing for Ceftazidime–avibactam (CZA) is 0.94 g IV every 24 to 48 h in patients with creatinine clearance <15 mL/min. However, there are no specific recommendations for CZA dosing in patients utilizing renal replacement therapy (RRT) [19]. Those doses are lower than those recommended for ceftazidime when used alone. Furthermore, Wenzler et al., in a case report, administered a dose of 1.25 g IV every 8 h of CZA to treat MDR PSA BSI in a patient utilizing continuous veno-venous hemofiltration (CVVH). They reported that the free drug concentrations of both ceftazidime and avibactam remained above the minimum inhibitory concentration (MIC) of PSA throughout the 8-h dosing interval [2]. It is important to note that despite the use of a higher-than-recommended dose, the BSI persisted for 5 days [2]. At our institution, the recommended doses were similar to those reported in Wenzler’s case report. We administered a full dose (loading dose) for up to 24 h, followed by 1.25 g IV every 8 h for patients utilizing CVVH and 1.25 g maintenance daily doses for patients on IHD [11].

Wooley and colleagues reported that 90% of the C/T dose is removed from the blood after one hemodialysis session [4]. However, after the post-dialysis redistribution, the net removal of ceftolozane was 66% and tazobactam was 56%. Additionally, Carbonell and colleagues suggested that the elimination half-life of ceftolozane is around 5.3 h in patients undergoing continuous hemodiafiltration [20]. The data suggest using a loading dose of 3 g followed by 1.5 g IV every 8 h to achieve a free drug concentration over time (*f*T>MIC) of 100%, which is associated with favorable pharmacodynamic outcomes in patients receiving continuous renal replacement therapy (CRRT) [19,21]. However, the FDA-approved dosing for patients utilizing IHD ranges from 0.75 to 2.25 g for the loading dose, followed by 0.15 to 0.45 g every 8 h based on the indication. Notably, there are no specific recommendations for patients utilizing CRRT, and these approved doses are lower than those suggested in other studies [8]. 

At our institution, we recommend a full dose of 3 g IV every 8 h for up to 24 h as a loading dose, followed by 0.75 g IV every 8 h for patients on IHD and 1.5 g IV every 8 h for those on CVVH. These doses were determined based on the pharmacokinetics of piperacillin/tazobactam in this population, where the minimum tazobactam dose used was 0.5 g for patients on CVVH and 0.25 g for those on IHD [10]. Further pharmacokinetic and clinical outcome studies are needed to determine the most effective doses of both CZA and C/T to achieve microbiologic and clinical cures in this population.

Our study has several limitations. Firstly, its retrospective nature introduces inherent biases. Additionally, being a single-center study limits the generalizability of our results. The study sample size included all cases meeting the inclusion criteria. While it may not compare to larger clinical trials, it is generally adequate and meaningful given the specific study criteria. Furthermore, due to the retrospective nature of the study, we could not measure drug levels to link the pharmacokinetics of these drugs with clinical outcomes. We also did not follow up with patients who had repeated cultures to assess the development of resistance to any of the study drugs. Finally, we did not document the time to start antibiotics, which could have impacted the outcomes. However, to our knowledge, this is the first study to address the clinical outcomes of these two new beta-lactam/beta-lactamase inhibitors in the described population.

## 4. Materials and Methods

We conducted a retrospective study at our hospital between September 2018, and March 2022. We included adults who received any of the study drugs for at least 3 days. Data collection from electronic medical records commenced after approval from the Research Ethics Committee. Informed consent was waived due to the retrospective nature of the study. We collected patients’ demographics, baseline characteristics, and infection-related data. This included infection parameters, culture results, antimicrobial use, doses and duration of therapy, PSA cultures, minimum inhibitory concentrations (MIC) of both drugs, and dialysis modalities.

Our primary endpoint was to determine clinical cure, as documented by the treating physician, in patients undergoing renal replacement therapy (RRT) (intermittent hemodialysis (IHD) or continuous veno-venous hemofiltration (CVVH)) treated with ceftazidime–avibactam (CZA) or ceftolozane–tazobactam (C/T). Clinical cure was defined as the resolution of fever, symptoms, and normalization of laboratory markers. Secondary endpoints included microbiologic cure (when repeat microbiology was obtained), 30-day recurrence, 30-day crude mortality, and documentation of central nervous system toxicity if present.

### 4.1. Patient Selection

Patients were included if they were adults (≥18 years of age), admitted to our hospital during the study period, diagnosed with active MDR PSA infection, required treatment with (CZA) or (C/T) at the clinicians’ discretion for either BSI or pneumonia, and underwent either intermittent hemodialysis (IHD) or continuous veno-venous hemofiltration (CVVH) during the therapy period. Patients were excluded if they received any of the study drugs for less than 3 days or if they were diagnosed with microbiological colonization rather than infection.

### 4.2. Definitions of Variables

The diagnosis of infections caused by MDR PSA was established based on combined clinical and microbiological criteria. Clinically, patients presented signs and symptoms consistent with an active infection (fever, elevated white blood cell count, elevated inflammatory markers, radiographic evidence of pneumonia, etc.). The etiological role of PSA was confirmed through positive cultures from normally sterile sites (blood for BSI, sputum, or bronchoalveolar lavage fluid in the case of pneumonia). These cultures were processed using standard microbiological techniques and the identification of PSA was performed using automated systems. To confirm the MDR status of the isolates, antimicrobial susceptibility testing was conducted according to CLSI guidelines. Strains were classified as MDR if they were resistant to at least three classes of antibiotics typically effective against PSA. Insignificant or contaminant results were excluded based on the clinical context and the source of the culture. For example, in the case of respiratory cultures, the presence of PSA had to be accompanied by a compatible clinical presentation and radiological findings to be considered significant. Colonization without signs of active infection was not included in the study. BSI was defined based on positive blood cultures for PSA accompanied by clinical signs and symptoms of infection, such as fever, chills, hypotension, and an elevated white blood cell count. Pneumonia was determined by signs and symptoms, physical exams, chest X-rays, and microbiological tests. Clinical cure was defined as the resolution of infection signs and symptoms as reported in the treating physician’s notes. Clinical failure was defined as either the persistence of functional symptoms for more than 72 h after initiating the study drugs (CZA or C/T) or as documented failure by the treating physician requiring a change in antimicrobials. Efficacy parameters included the clinical and microbiological clearance of the bacteria, as well as changes in inflammatory markers (white blood cells [WBCs], C-reactive protein [CRP], and procalcitonin). Recurrence was defined by repeated positive culture of the same pathogen with clinical symptoms in the 30-day follow-up period after the resolution of the initial episode. The duration of therapy was calculated from the initiation of C/T or CZA, regardless of any empirical use of other antibiotics. This approach aligns with hospital policy, which mandates the use of C/T or CZA when they are the sole susceptible agents.

### 4.3. Renal Replacement Therapies

At our institution, we offer both intermittent hemodialysis (IHD) and continuous veno-venous hemofiltration (CVVH) modalities for renal replacement therapy. Critically ill patients may transition between these modalities based on their hemodynamic stability. For CVVH, the hemofiltration replacement fluid rate ranges from 20 to 25 mL/kg/h, with an average net ultrafiltration rate of 50–200 mL/h as tolerated. The median blood flow rate is maintained at 180 to 200 mL/min. Patients may receive no anticoagulation, heparin, or citrate as anticoagulants. The median duration of CVVH treatment is approximately 20 h per day, accounting for any interruptions. Most patients receive replacement fluids at a ratio of 70% prefilter and 30% postfilter. The Prismaflex^®^ M150 filter (Baxter Healthcare Corporation, Deerfield, IL, USA) is predominantly used, with the M100 filter used to a lesser extent.

In IHD, sessions typically last between 3.5 and 4 h and are conducted 3 to 4 times weekly. The blood flow rate ranges from 250 to 400 mL/min, the dialysate flow rate ranges from 500 to 600 mL/min, and the ultrafiltration rate ranges from 500 to 3500 mL per treatment session, depending on the clinical scenario. The Polyflux^®^ 140 H and 170 H filters (Baxter Healthcare Corporation, Deerfield, IL, USA) are the most commonly used in hemodialysis.

### 4.4. Data Analysis

The data are presented as the median (IQR) due to the small sample size. Categorical variables are expressed as proportions. The Mann–Whitney U test was used to compare values between independent groups. Discrete data were analyzed using the χ^2^ test or Fisher exact test for small numbers. Multivariate logistic regression analysis was conducted to identify independent risk factors for clinical cure. Variables that were clinically relevant or known to impact clinical cure were included in the regression analysis based on the results of the univariate analysis. The goodness-of-fit was assessed using the Hosmer–Lemeshow test. Statistical significance was set at a two-sided *p*-value < 0.05. All statistical analyses were performed using SPSS statistical package version 28 for Microsoft Windows (IBM, Armonk, NY, USA).

## 5. Conclusions

Our findings indicated that C/T and CZA did not significantly differ in achieving a clinical cure in patients with MDR PSA infections undergoing RRT. Additionally, patients with BSI secondary to MDR PSA treated with either CZA or C/T had higher odds of achieving clinical cure compared to those with pneumonia. However, larger clinical trials are needed to confirm these results.

## Figures and Tables

**Table 1:** Demographics and baseline characteristics of the study patients.

Variable	Ceftazidime–Avibactam (*n* = 25)	Ceftolozane–Tazobactam (*n* = 20)	*p*-Value
Gender (male), *n* (%)	9 (36)	7 (35)	0.944
Age, years	69 (52–81)	69 (61.5–83)	0.599
Weight, kg	70 (55.5–81.5)	66 (57–79)	0.732
Body Mass Index, kg/m^2^	25 (22–31)	26 (22–30.5)	0.873
Comorbidities, *n* (%)			
Hypertension	19 (76)	14 (70)	0.651
Diabetes Melius	16 (64)	14 (70)	0.671
Congestive Heart Failure	6 (24)	6 (30)	0.651
Coronary artery disease	10 (40)	8(40)	1
Dyslipidemia	9 (36)	7 (35)	0.944
Liver Disease	3 (12)	1 (5)	0.394
Cerebrovascular Accident	9 (36)	11 (55)	0.202
Respiratory Failure	21 (84)	15 (75)	0.352
Diagnosis, *n* (%)			0.358
Pneumonia	19 (76)	17 (85)	
Blood stream infection	6 (24)	3 (15)	
Antibiotic Initiation Unit, *n* (%)			0.883
Intensive Care Unit	18 (72)	14 (70)	
Acute Care Unit	7 (28)	6 (30)	

Data are expressed as median and (25–75 percentiles) or count and percentage. *n* = Number of patients who received this drug.

**Table 2:** Treatment outcomes.

Variable	Ceftazidime–Avibactam (*n* = 25)	Ceftolozan–Tazobactam (*n* = 20)	*p*-Value
Clinical Cure, *n* (%)	12 (48%)	12 (60%)	0.423
Duration of therapy, day	6 (4–8)	7 (4–11.5)	0.264
Carbapenem resistant, *n* (%)	18 (75)	18 (90)	0.187
Received a loading dose, *n* (%)	18 (72)	15 (75)	0.821
Concomitant antibiotics, *n* (%)	15 (60)	14 (70)	0.486
WBC count at diagnosis, cells/mm^3^	14.2 (11–27)	18.1 (13.5–26)	0.882
Days for WBC count to normalize, day	3 (1–6)	3.5 (1.75–10.75)	0.535
Temperature at therapy initiation, °C	36.9 (36–37.6)	36.3 (36–37.7)	0.33
CRP Bassline, mg/L	110 (57–239)	231 (70–298)	0.43
CRP at end of therapy, mg/L	53 (27–89)	90 (36–138)	0.283
Procalcitonin Baseline, mcg/L	1 (0.7–2)	1.7 (0.5–6.3)	0.512
Procalcitonin at end of therapy, mcg/L	1.9 (0.6–3.1)	2.1 (1.5–2.4)	0.295
Vasopressors used, *n* (%)	8 (32)	7 (35)	0.832
Mechanical Ventilation, *n* (%)	13 (52)	10 (50)	0.894
RRT Indication, *n* (%)			0.126
AKI	23 (92)	15 (75)	
ESKD	2 (8)	5 (25)	
30-day recurrence, *n* (%)	3 (12)	6 (30)	0.082
30-day Mortality, *n* (%)	13 (52)	10 (50)	0.894

WBC, white blood cells; CRP, C reactive protein; RRT, renal replacement therapy; AKI, acute kidney injury; ESKD, end-stage kidney disease. Data are expressed as median and (25–75 percentiles) or count and percentage.

**Table 3:** Multivariate logistic regression analysis to determine the risk factors associated with clinical cure.

Variables	OR (95% CI)	*p*-Value
BSI (pneumonia reference)	25 (1.63–411.7)	0.021
C/T (CZA reference)	2.8 (0.44–18)	0.269
Concomitant antibiotics	3.4 (0.47–25)	0.223
Loading dose/day (g)	1.02 (0.58–1.77)	0.954
Maintenance dose/day (g)	0.76 (0.25–2.3)	0.622
Duration of therapy (days)	1.08 (0.94–1.24)	0.276

The Hosmer–Lemeshow’s test *p* value = 0.098; BSI = Blood stream infection; CZA = ceftazidime–avibactam; C/T = ceftolozane–tazobactam.

## Data Availability

The original contributions presented in the study are included in the article, further inquiries can be directed to the corresponding author. Available upon reasonable request, will need IRB approval to share based on the hospital policy.
